# Enhanced uptake of multiple sclerosis-derived myelin by THP-1 macrophages and primary human microglia

**DOI:** 10.1186/1742-2094-11-64

**Published:** 2014-03-31

**Authors:** Debbie AE Hendrickx, Karianne G Schuurman, Michael van Draanen, Jörg Hamann, Inge Huitinga

**Affiliations:** 1 Neuroimmunology Research Group, Netherlands Institute for Neuroscience, Meibergdreef 47, 1105 BA Amsterdam, The Netherlands; 2 Department of Experimental Immunology, Academic Medical Center, Amsterdam, The Netherlands

**Keywords:** Multiple sclerosis, Demyelination, Microglia, Phagocytosis

## Abstract

**Background:**

The pathological hallmark of multiple sclerosis (MS) is myelin phagocytosis. It remains unclear why microglia and macrophages demyelinate axons in MS, but previously found or yet-unknown changes in the myelin of MS patients could contribute to this process. We therefore studied whether myelin from normal-appearing white matter (NAWM) of MS donors is phagocytosed more efficiently than myelin from control donors.

**Methods:**

Myelin was isolated from 11 MS and 12 control brain donors and labeled with the pH-sensitive fluorescent dye pHrodo to quantify uptake in lysosomes. Phagocytosis by differentiated THP-1 macrophages and by primary human microglia was quantified with flow cytometry. Whereas myelin uptake by THP-1 macrophages reached a plateau after approximately 24 hours, uptake by primary human microglia showed an almost linear increase over a 72–hour period. Data were statistically analyzed with the Mann–Whitney *U* test.

**Results:**

MS-derived myelin was phagocytosed more efficiently by THP-1 macrophages after 6-hour incubation (*P* = 0.001 for the percentage of myelin-phagocytosing cells and *P* = 0.0005 for total myelin uptake) and after 24-hour incubation (*P* = 0.0006 and *P* = 0.0001, respectively), and by microglia after 24-hour incubation (*P* = 0.0106 for total myelin uptake). This enhanced uptake was not due to differences in the oxidation status of the myelin. Interestingly, myelin phagocytosis correlated negatively with the age of myelin donors, whereas the age of microglia donors showed a positive trend with myelin phagocytosis.

**Conclusions:**

Myelin isolated from normal-appearing white matter of MS donors was phagocytosed more efficiently than was myelin isolated from control brain donors by both THP-1 macrophages and primary human microglia. These data indicate that changes in MS myelin might precede phagocyte activation and subsequent demyelination in MS. Identifying these myelin changes responsible for enhancing phagocytic ability could be an interesting therapeutic target to prevent or inhibit formation or expansion of MS lesions. Moreover, during aging, microglia enhance their phagocytic capacity for myelin phagocytosis, but myelin reduces its susceptibility for uptake.

## Introduction

Demyelination is the pathologic hallmark of multiple sclerosis (MS) [1], but the initial cause that triggers resident microglia and infiltrating macrophages to start phagocytosing myelin remains to be elucidated. Many studies focused on phagocytic cells to stop them as effector cells in demyelination [2], but relatively little research focused on the myelin itself.

Different studies have reported altered myelin oxidation in multiple sclerosis. Lipid peroxidation-derived malondialdehyde (MDA) was increased in and around active MS lesions [3], and rat MDA-modified myelin oligodendrocyte glycoprotein (MOG) was phagocytosed more efficiently than was naïve MOG via scavenger receptor (SR) class A on mouse macrophages [4]. 4-Hydroxynonenal, another product of lipid peroxidation, was increased in myelin isolated from normal-appearing white matter (NAWM) of MS donors compared with myelin isolated from control donors [5]. Lipid peroxidation might create new ligands for SRs that are known to bind oxidized low-density lipoproteins (oxLDLs) and oxidized phospholipids on apoptotic cells [6,7]. In line herewith, SRs have been suggested to play a role in myelin phagocytosis [4,8-12] or the animal model of multiple sclerosis, experimental autoimmune encephalomyelitis (EAE) [13,14]. Myelin lipid composition is also altered in NAWM of MS donors, and autoantibodies to several myelin lipids were elevated in sera of MS donors (reviewed in [15]).

Besides myelin lipids, myelin proteins are also subject to changes in MS. Myelin basic protein (MBP) isolated from NAWM of MS donors is overall less phosphorylated and more methylated and citrullinated than MBP of control donor WM [16]. These posttranslational modifications alter membrane stability, making MBP more susceptible for proteases, which might eventually create neo-self antigens that induce disease. Increased activity of peptidylarginine deiminases (PADs), enzymes generating citrulline, and increased protein citrullination was found in NAWM of MS donors [17]. 2-Chloroacetamidine, a PAD inhibitor, reduced PAD activity in human brain extracts and attenuated disease in four different demyelinating mouse models, indicating that citrullination determines severity of disease. However, no anti-citrullinated protein antibodies were found in serum of MS patients [18], indicating that citrullination itself is not a direct target for autoimmunity in multiple sclerosis.

Opsonization might also mark myelin for phagocytosis. Expression of the complement factors C3d, C4d, and C3b was enhanced on myelin of MS lesions and often colocalized with HLA^+^ macrophages [19,20]. Complement opsonization is known to increase myelin phagocytosis *in vitro*[10-12,21]. Myelin-directed autoantibodies can also opsonize myelin and increase its uptake *in vitro*[10,21,22].

Finally, heat-shock protein 70 (hsp70) was found to be associated with MBP and proteolipid protein (PLP) in chronic active MS lesions and association with MBP increased uptake and presentation by different phagocytes *in vitro*[23]. Moreover, hsp70 directly enhanced the phagocytic capacity of macrophages [24], and the small heat-shock protein αB-crystallin was identified as immunogenic protein in multiple sclerosis-affected myelin [25] and in NAWM of preactive lesions of MS patients [26].

We hypothesized that the changes found in MS myelin could increase its uptake compared with myelin from controls without neurologic abnormalities. To test this hypothesis, we isolated myelin from NAWM of 11 MS and 12 control donors immediately postmortem and quantified its phagocytosis by macrophages and microglia. Myelin was labeled with pHrodo, a pH-sensitive dye used previously to detect engulfment of apoptotic cells [27]. The pHrodo-conjugated myelin allowed us to quantify material taken up into the acidic milieu of the phagolysosomes while excluding material attached to the cell surface. We showed that MS myelin is phagocytosed more efficiently by macrophages derived from the human monocytic cell line THP-1 and by primary human microglia, indicating that aforementioned or yet-unknown changes within MS myelin can trigger phagocytosis.

## Materials and methods

### Human brain tissue

Postmortem human brain tissue was provided by the Netherlands Brain Bank (NBB, Amsterdam, The Netherlands). Permission was obtained from donors for brain autopsy and the use of tissue and clinical information for research purposes. At autopsy, the corpus callosum and/or subcortical white matter was dissected and stored in Hibernate A medium (Brain Bits LLC, Springfield, IL, USA) until further processing. The absence of lesions in MS NAWM was confirmed by scanning brain slices with T_2_ or 3D-FLAIR MRI [28]. Myelin was isolated from 11 MS and 12 donors without neurologic abnormalities (Table 1). The mean age of MS donors was 62.6 years (range, 50 to 73 years), and for control donors, 80.2 years (range, 60 to 94 years). The mean postmortem delay (PMD) was 8:47 hours for MS donors (range, 7:05 to 10:40 hours) and 6:13 hours for control donors (range, 4:15 to 8:35 hours). The mean pH of the cerebrospinal fluid (CSF) was 6.46 for MS donors (range, 6.16 to 7.10) and 6.51 for control donors (range, 6.03 to 7.07).

**Table 1:** Detailed information of myelin donors

**NBB**	**F/M**	**Age**	**PMD**	**pH**	**Diagnosis**	**Disease duration**	**Disease course**	**Treatment last 3 months**
2010-005	F	68	10:40	6.4	MS	57	RR-SP	
2010-045	F	84	07:35	-	MS	50	PR	
2010-117	F	60	10:40	6.48	MS	7	SP	
2011-008	M	54	08:15	6.39	MS	12	PP	
2011-035	F	50	07:35	6.45	MS	17	SP	
2011-048	M	53	10:00	6.38	MS	24	SP	
2011-080	F	56	08:25	6.16	MS	32	SP	Vitamin D
2011-089	M	64	07:30	6.49	MS	35	RR-SP	
2011-093	M	56	10:10	7.1	MS	13	RR	
2011-100	F	71	07:05	6.32	MS	31	RR-SP	
2011-120	M	73	08:45	6.4	MS	42	SP	β-interferon
2010-007	F	85	05:20	7.07	HC	-	-	
2010-062	F	94	05:50	7.04	HC	-	-	
2010-068	M	85	08:35	6.04	HC	-	-	
2010-070	F	60	07:30	6.8	HC	-	-	
2011-021	F	85	07:05	-	HC	-	-	
2011-039	F	91	04:15	6.5	HC	-	-	
2011-046	F	89	04:45	6.67	HC	-	-	
2011-049	F	83	04:40	6.04	HC	-	-	
2011-091	M	76	06:45	6.31	HC	-	-	
2012-048	M	81	06:40	6.7	HC (depression)	-	-	
2012-049	F	70	07:35	6.03	HC	-	-	
2012-052	F	64	05:40	6.35	HC	-	-	

F/M, female/male; HC, healthy control; MS, multiple sclerosis; NBB, Netherlands Brain Bank donor number; PMD, postmortem delay; PP, primary progressive MS; RR, relapsing remitting MS; SP, secondary progressive MS.

Microglia were isolated from different donors (five controls, three with Parkinson disease, one with MS, and one with frontotemporal dementia) (Table 2). The mean age was 72.8 years (range, 57 to 89 years); the mean PMD was 6:07 (range, 3:45 to 10:45 years), and the mean CSF pH was 6.51 (range, 6.2 to 6.73).

**Table 2:** Detailed information of microglia donors

**NBB**	**F/M**	**Age**	**PMD**	**pH**	**Diagnosis**
2012-071	F	57	7:40	6.47	HC
2012-080	M	86	5:50	6.48	PD
2012-091	F	61	5:55	6.63	PD
2012-101	M	80	4:25	6.59	HC
2012-104	M	79	6:30	6.3	HC (personality disorder)
2012-113	M	59	10:45	6.5	MS
2013-003	M	60	5:00	6.2	PD
2013-008	F	74	3:45	6.55	FTD
2013-010	F	89	6:35	6.73	HC
2013-016	M	83	5:15	6.6	HC

F/M, female/male; FTD, frontotemporal dementia; HC, healthy control; MS, multiple sclerosis; NBB, Netherlands Brain Bank donor number; PD, Parkinson disease; PMD, postmortem delay.

### Microglia isolation

Microglia isolation was performed as described previously [29,30]. In brief, tissue was mechanically and enzymatically dissociated, and erythrocytes were lysed. Cells, myelin, and debris were then separated by density gradient separation by using Percoll (GE Healthcare, Uppsala, Sweden) of ρ = 1.03 and ρ = 1.095 and buffer. Myelin was either discarded or collected from the buffer and Percoll ρ = 1.03 interlayer and stored until further processing. Cells were collected from the Percoll ρ = 1.03 and ρ = 1.095 interlayer and counted. Microglia were further isolated by magnetic bead sorting (Miltenyi Biotec GmbH, Bergisch Gladbach, Germany) by using CD15 beads for negative selection to remove granulocytes and subsequent CD11b beads for positive selection of microglia. The purity of the isolated microglia and their activation stage were assessed with flow cytometry (see later). Microglia were cultured in RPMI glutaMAX medium containing 10% fetal calf serum (FCS) and 1% penicillin/streptomycin (all Invitrogen, Carlsbad, CA, USA) and allowed to adhere before myelin incubation.

### Myelin isolation and labeling

The myelin-containing fraction was collected after Percoll gradient separation (see Microglia isolation) and further purified by a sucrose gradient, as described previously [31]. The myelin fraction was washed thrice in 0.32 *M* sucrose (Sigma-Aldrich, St. Louis, MO, USA), resuspended in 0.32 *M* sucrose, and underlain with 0.85 *M* sucrose. This gradient was centrifuged for 50 minutes at 4,500 *g*. Myelin was collected from the interface and washed in water to induce an osmotic shock to remove any remaining cells. After centrifugation, myelin was collected, resuspended in PBS, and stored at -80°C until further processing.

Myelin concentration was measured with the bicinchoninic acid (BCA) protein assay kit (Pierce Thermo Scientific, Rockford, IL, USA). For all samples, endotoxin content was measured with the Toxin sensor LAL endotoxin assay kit (Genscript USA Inc, Piscataway, NJ, USA). Most samples stayed under the detection limit of 0.05 EU/ml, and the small amount of endotoxin (0 to 0.019 EU/ml per μg myelin) found in some samples did not correlate with myelin phagocytosis. Myelin was labeled with the pH-sensitive dye pHrodo red, succinimidyl ester (Invitrogen), which binds to proteins, to visualize uptake in the lysosomal compartment [27]. The pHrodo dye was dissolved in 1 ml dimethyl sulfoxide (DMSO) and used 1:100 per milligram myelin. Myelin was resuspended in PBS, pH 8, and incubated with pHrodo for 45 minutes at RT. Labeled myelin was spun down and resuspended in PBS pH 7.4 and stored at -80°C. The labeling efficiency of individual myelin samples was measured by resuspending labeled myelin in PBS with pH 4 and measuring fluorescence on a Varioskan Flash (excitation, 530, and emission, 560/585 nm).

### Cell culture and phagocytosis assay

The human monocytic cell line THP-1 was cultured in RPMI glutaMAX medium containing 10% FCS and 1% penicillin/streptomycin. Cells were cultured in plates coated with poly (2-hydroxyethyl methacrylate), otherwise known as hydron (Sigma-Aldrich), to prevent adherence. Cells were differentiated into macrophage-like cells by stimulation with 160 n*M* phorbol myristate acetate (PMA) for 24 hours, followed by another 24-hour culture in normal medium.

For phagocytosis assays, THP-1 macrophages were incubated with 12.5 μg pHrodo-labeled myelin per 80,000 cells for either 6 or 24 hours. Microglia were incubated with 1 μg pHrodo-labeled myelin per 90,000 cells for 24 hours. After incubation, cells were collected (THP-1 macrophages were free floating, and microglia were detached by 5-minute incubation in 25 m*M* EDTA at 37°C) and washed in cold PBS with 1% BSA and stained for flow-cytometric analysis.

### Flow cytometry

Primary microglia were stained immediately after isolation with anti-human CD11b-PE (clone ICRF44; eBioscience, San Diego, CA, USA) and anti-human CD45-FITC (clone HI30; Dako, Glostrup, Denmark) for 30 minutes on ice to assess their initial activation status *ex vivo*. The viability marker 7-amino-actinomycin D (7-AAD; BD Biosciences) was added approximately 10 to 15 minutes before analysis on the FACScalibur machine (BD Bioscience).

For the quantification of myelin uptake, THP-1 macrophages and microglia were collected as described before and incubated 1:2,000 with the viability dye eFluor 780 (eBiosciences) for 30 minutes on ice. Uptake of pHrodo-labeled myelin was measured on a FACSCanto machine (BD Biosciences), by using identical settings for either THP-1 or microglia, and analyzed by using FlowJo 7.6 software. Phagocytosis was expressed as percentage of live cells that took up myelin and as geomean fluorescence intensity (geoMFI) of the pHrodo signal, indicating the total amount of myelin phagocytosed. Doublets were excluded in the microglia phagocytosis assays.

### TBARS (lipid peroxidation) assay

Lipid peroxidation of the myelin samples was determined by measuring MDA in a thiobarbituric acid-reactive substances (TBARS) assay. The 200 μg myelin was resuspended in 100 μl PBS, pH 7.4, and incubated with 200 μl ice-cold 10% trichloroacetic acid (TCA; VWR International, Lutterworth, England) for 15 minutes on ice to precipitate protein. Then, 300 μl 0.67% 2-thiobarbituric acid (TBA) in 10% DMSO was added, and samples were incubated at 99°C on a heat plate for 15 minutes. After incubation, samples were spun down at 2,200 *g* for 15 minutes at 4°C and left to cool. Fluorescence (excitation 530 and emission 550 nm) was measured in duplo against an MDA standard (ranging from 50 p*M* to 5 n*M*; Sigma-Aldrich) on a Varioskan Flash (ThermoScientific, Waltham, MA, USA).

### Image acquisition

Fluorescent images were taken on an AxioVert microscope (Zeiss, Oberkochen, Germany) with Neoplanfluor objectives by using an Exi Aqua Bio-imaging microscopy camera (QImaging, Surrey, BC, Canada) and ImagePro software (MediaCybernetics, Bethesda, MD, USA).

### Statistical analysis

Statistical analysis was performed by using GraphPad Prism version 5 software (GraphPad Inc., La Jolla, CA, USA). Differences between individual samples were assessed with the Mann–Whitney *U* test. Correlations between two continuous parameters were assessed with the Spearman correlation coefficient. *P* values <0.05 were considered significant.

## Results

### Analysis of pHrodo-labeled myelin internalization by THP-1 macrophages and primary human microglia

**Figure 1:**
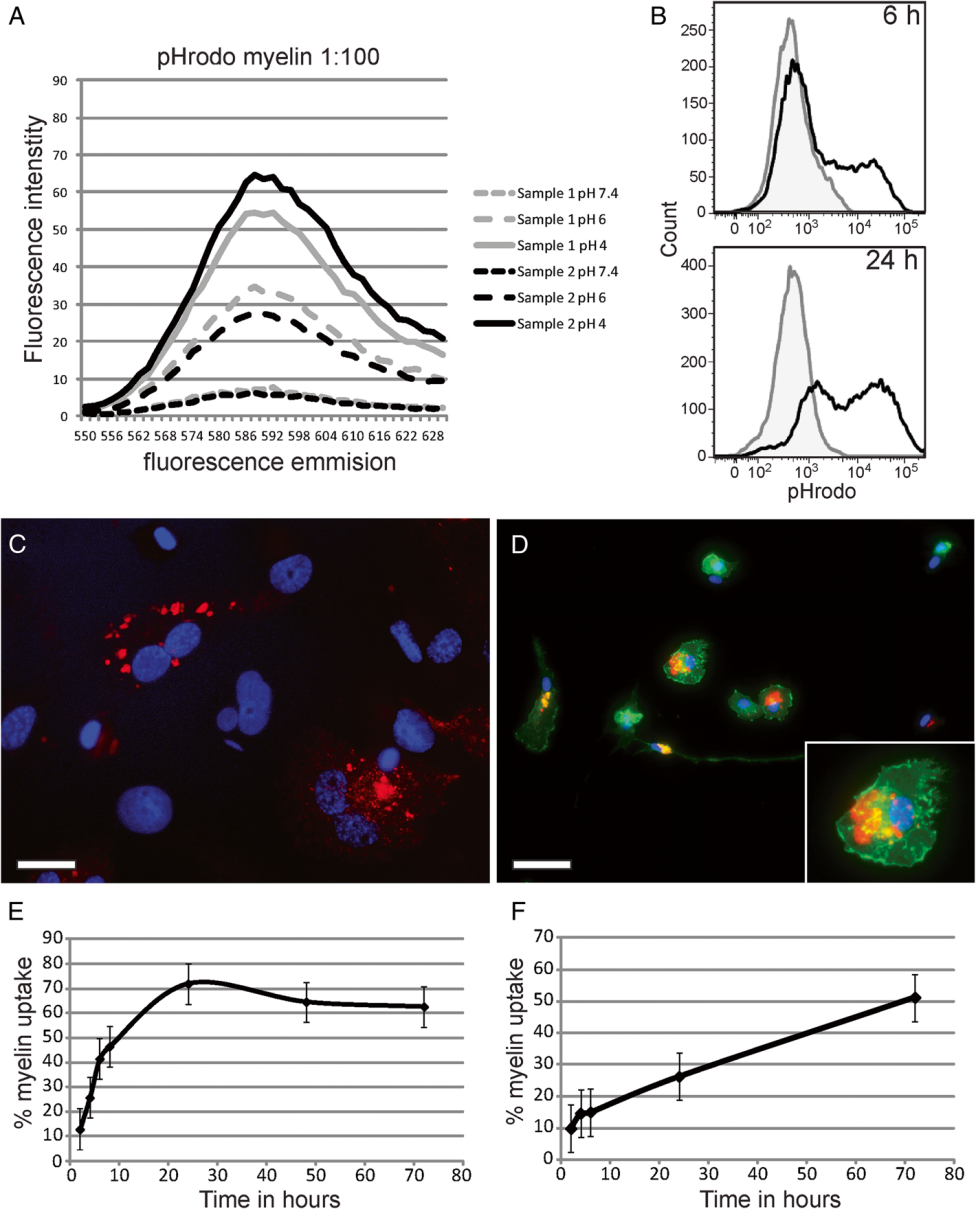
**Quantification of myelin phagocytosis by using a pH-sensitive fluorescent dye. (A)** Myelin was labeled with pHrodo and resuspended in PBS pH 7.4, 6, or 4. Fluorescent intensity was measured on a Varioskan Flash and increased in signal with decreasing pH. **(B)** THP-1 macrophages were incubated without (gray line) or with 12.5 μg pHrodo-labeled myelin (black line) for 6 and 24 hours, and measured with flow cytometry. Fluorescence increased over time as THP-1 macrophages phagocytosed myelin. Uptake of pHrodo-labeled myelin (red) by **(C)** THP-1 macrophages and by **(D)** primary human microglia. Microglia were stained with phalloidin (green) to visualize actin filaments. Insert shows a close-up of a phagocytosing microglia. **(E)** Percentage of cells that phagocytosed pHrodo-labeled myelin in **(E)**. THP-1 macrophages and **(F)** primary human microglia over time measured with flow cytometry. Scale bar is 25 μm.

Myelin conjugated with the pH-sensitive dye pHrodo emitted a red fluorescent signal as pH decreased (Figure 1A), which enabled detection of its uptake into the acidic milieu of the phagolysosomes by flow cytometry. This method has already been successfully used to study the uptake of apoptotic cells [27]. pHrodo itself did not have any effect on the phenotype of THP-1 macrophages (data not shown). A distinct pHrodo-positive cell population formed when THP-1 macrophages were incubated with pHrodo-labeled myelin for 6 and 24 hours compared with control cells (Figure 1B). The shift in fluorescent signal of the pHrodo-negative population compared with control cells after 24 hours is due to a decrease in pH of the medium, causing the bound nonphagocytosed myelin slightly to increase fluorescence (about 135% as measured on a Varioskan Flash). Phagocytosed pHrodo-labeled myelin was clearly visible in vesicles in both THP-1 macrophages (Figure 1C) and in primary human microglia (Figure 1D). In contrast, myelin that was not phagocytosed, visualized by bright field, was not or was barely fluorescent (see Additional file 1).

Next, the optimal incubation time for quantifying myelin uptake by THP-1 macrophages (Figure 1E) and primary human microglia (Figure 1F) was determined. THP-1 macrophages were incubated with pHrodo-labeled myelin (from donors with different neurologic backgrounds) for 2, 4, 6, 8, 24, 48, and 72 hours, and the percentage of cells that phagocytosed myelin was measured with flow cytometry. As shown, phagocytosis peaked at 24 hours, after which a plateau was reached. This indicates that either THP-1 does not phagocytose more myelin or an equilibrium is reached between uptake and breakdown. For further experiments, THP-1 macrophages were incubated for 6 hours and for 24 hours, to measure progressing and peak phagocytosis, respectively. Microglia were incubated with pHrodo-labeled myelin (from donors with different neurologic backgrounds) for 2, 4, 6, 24, and 72 hours. Contrary to THP-1 macrophages, myelin uptake by microglia was a continuous process that did not saturate within 72 hours, the latest time point measured. However, because of increased cell death at this time point, phagocytosis was measured after 24 hours in further experiments. The time dynamics of myelin phagocytosis (pHrodo signal) during pilot studies was the same in either THP-1 macrophages or human microglia, independent of the neurologic background of the myelin donors. This indicates that differences found in uptake of MS and control donor myelin at specific time points is not a result of differences in phagocytosis speed or endosomal acidification, as this would result in a shift in the timeline experiments.

The fluorescent intensity of individual myelin samples was measured at pH 4. Labeling efficiency varied slightly between the myelin samples used, but no correlation was found between the fluorescence of myelin samples at pH 4 and the fluorescence intensity of cells that had taken up these myelin samples (percentage of fluorescent cells and total amount of myelin uptake expressed as the geoMFI), indicating that labeling efficiency did not influence the phagocytosis results (not shown).

### Increased phagocytosis of multiple sclerosis-derived myelin

**Figure 2:**
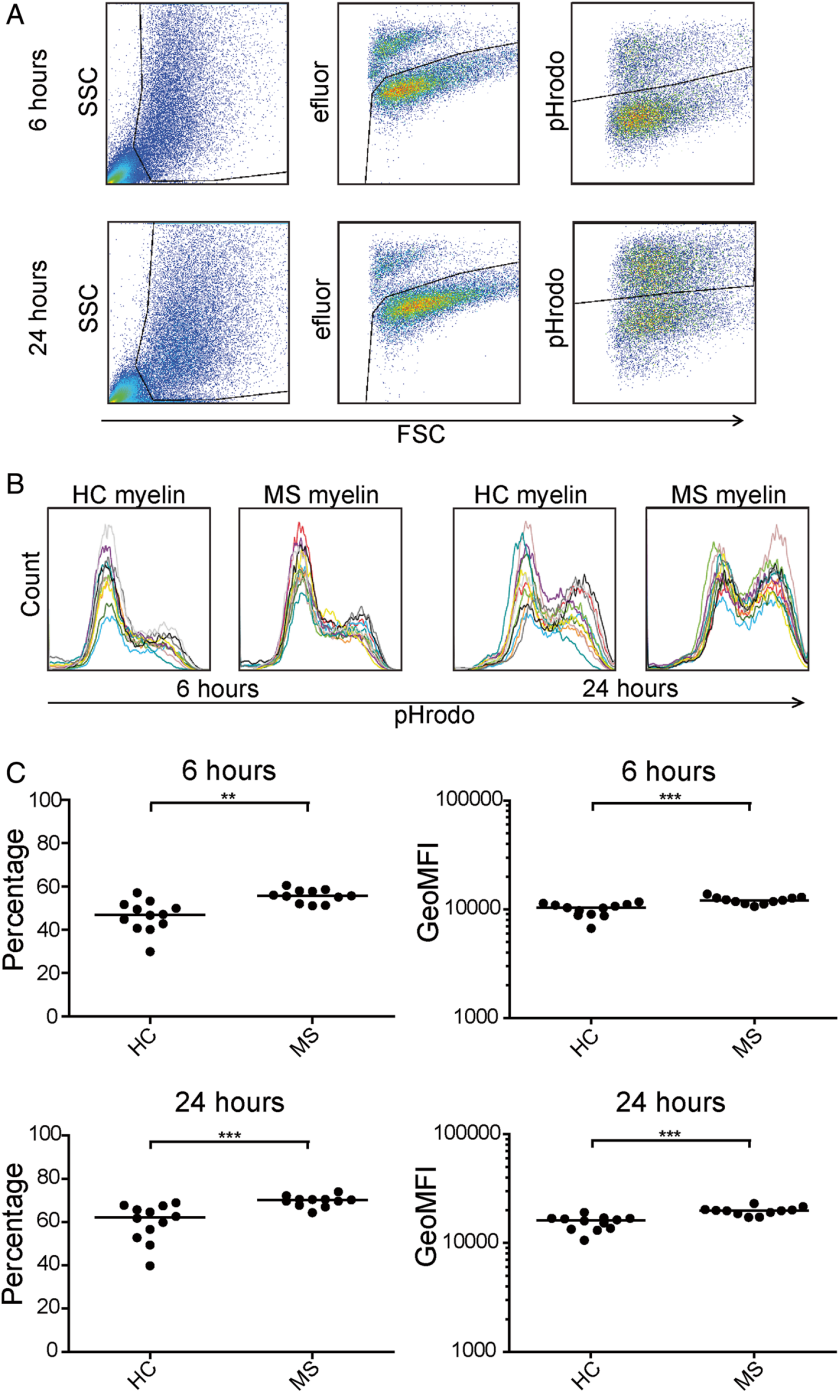
**Increased uptake of MS myelin by THP-1 macrophages after 6 and 24 hours. (A)** Flow-cytometric analysis of live cells defined by forward and sideward scatter (FSC and SSC) characteristics and the viability dye eFluor for uptake of pHrodo-labeled myelin after 6 and 24 hours of incubation. **(B)**. Histograms showing uptake of 11 MS and 12 control myelin samples (pHrodo signal) after 6 and 24 hours. **(C)** THP-1 macrophages phagocytosed MS myelin more efficiently than control myelin after 6 and 24 hours. Both the percentage of cells that took up myelin and the amount of myelin taken up (geoMFI) was significantly higher when cells were incubated with MS myelin. Shown is the mean for each myelin sample in four independent experiments. Data were analyzed with the Mann–Whitney *U* test. HC, healthy control; MS, multiple sclerosis. * < 0.05; ** < 0.005; *** < 0.0005.

THP-1 macrophages were incubated with pHrodo-labeled myelin samples from NAWM of 11 MS and 12 control donors for 6 hours and 24 hours, and phagocytosis by viable cells was measured by using flow cytometry (Figure 2A). At 6 hours and 24 hours, a shift toward more uptake of MS myelin was detected (Figure 2B). Both the percentage of cells that had phagocytosed myelin and the amount of myelin taken up by the cells were significantly higher when cells were incubated with MS myelin as compared with control myelin after 6 hours (*P* = 0.001 and *P* = 0.0005, respectively) and 24 hours (*P* = 0.0006 and *P* = 0.0001, respectively) (Figure 2C).

**Figure 3:**
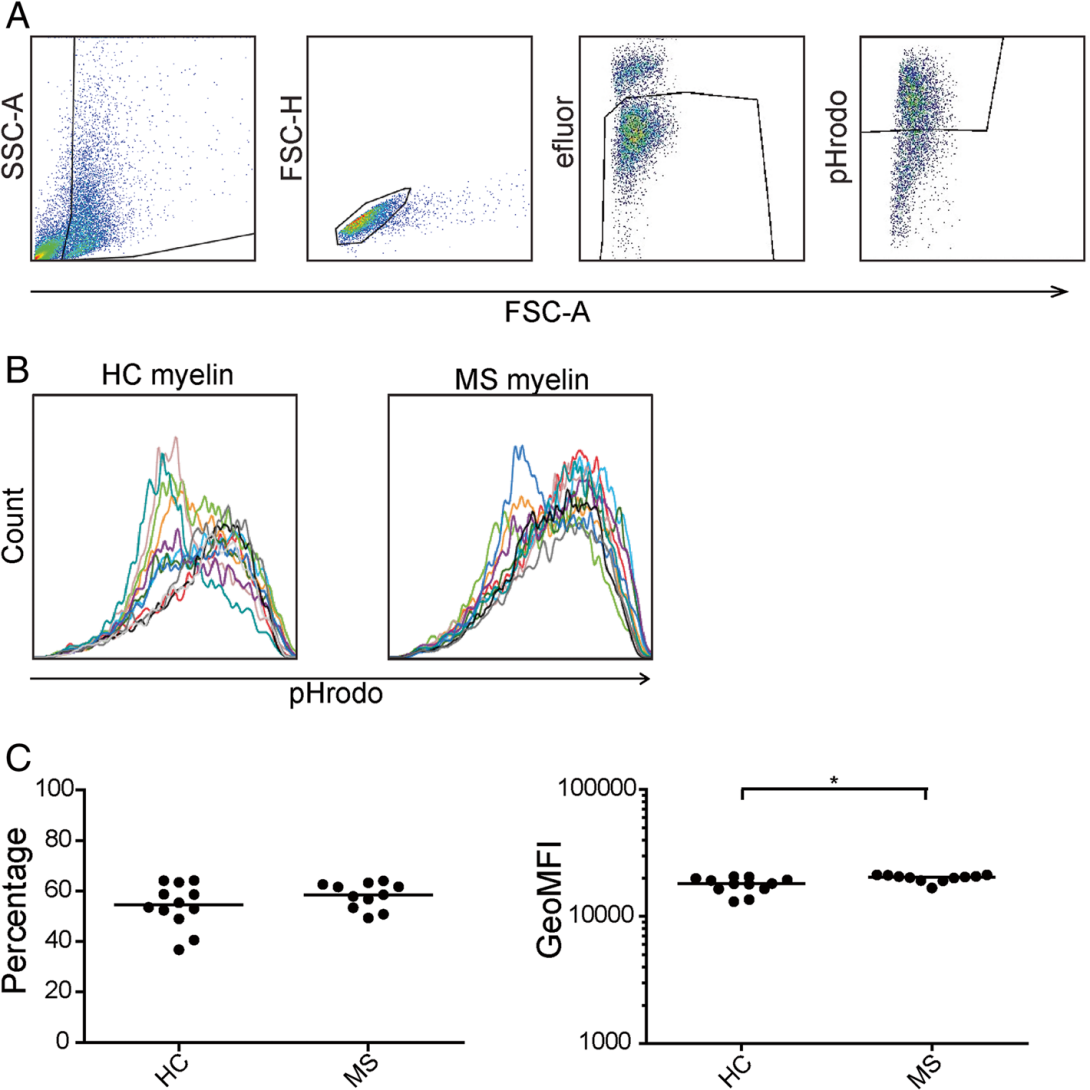
**Increased uptake of MS myelin by primary human microglia after 24 hours. (A)** Flow-cytometric analysis of live cells defined by forward and sideward scatter (FSC and SSC) characteristics and the viability dye eFluor were analyzed for uptake of pHrodo-labeled myelin after 24 hours of incubation. **(B)** Histograms showing uptake of 11 MS and 12 control myelin samples (pHrodo signal) after 24 hours. **(C)** Microglia phagocytosed MS myelin more efficiently than control myelin after 24 hours. The amount of myelin phagocytosed (geoMFI) was significantly higher when cells were incubated with MS myelin. Shown is the mean of 10 independent experiments. Data were analyzed with the Mann Whitney *U* test. HC, healthy control; MS, multiple sclerosis.

Next, we measured uptake of the myelin samples by primary microglia isolated from fresh human brain tissue. Microglia were isolated from NAWM of 10 brain donors and incubated for 24 hours with the 11 MS and 12 control myelin samples. Myelin phagocytosis by viable cells was assessed with flow cytometry (Figure 3A). Again, a shift was observed toward more uptake of MS myelin (Figure 3B). Quantification of the uptake of the individual myelin samples by the 10 microglia isolates revealed no significant difference in the percentage of pHrodo-positive cells (*P* = 0.4791). However, an increase was observed in the amount of myelin taken up (*P* = 0.0106) when cells were incubated with MS myelin as compared with control myelin (Figure 3C).

Because the microglia studied here were isolated from donors with different ages, we compared their phagocytosis capacity. We observed a trend toward an increase in myelin uptake (percentage and total amount) with donor age (see Additional file 2). Of note, the initial activation status of the isolated microglia, based on CD11b and CD45 expression analyzed with flow cytometry, did not correlate with myelin uptake (data not shown).

MDA, a measurement of lipid peroxidation, was not significantly different between myelin samples from MS and control donors, nor did it correlate with myelin phagocytosis in our experiments (data not shown).

### Effect of age and PMD of myelin donors on phagocytosis

Myelin was isolated as donors came to autopsy and was therefore not matched for age and PMD initially. Age (MS, 62.6 ± 10.4 years; controls, 80.2 ± 10.7 years) and PMD (MS, 8:47 ± 1:21 hours; controls, 6:13 ± 1:21 hours) differed significantly between MS and control donors (*P* = 0.0023 and *P* = 0.0008, respectively) (see Additional file 3A), due to earlier death of MS donors and the postmortem MRI performed on brain slices of MS patients. Because both age and PMD also correlated with myelin uptake (Additional file 3B and C), they could have influenced phagocytosis efficacy in our assays.

To correct for age and PMD retrospectively, we compared myelin phagocytosis between myelin samples matched for age or PMD (Additional file 3C). Within the age-matched subgroup, comprising six MS and nine control myelin samples, an increase was still observed in the percentage of MS myelin-phagocytosing THP-1 macrophages and total MS myelin uptake after 6 hours (*P* = 0.0120 and *P* = 0.0016, respectively) and 24 hours (*P* = 0.0004 and *P* = 0.0008, respectively) incubation (Table 3 and Additional file 3D). Similarly, increased uptake of MS myelin by THP-1 macrophages was observed in the PMD-matched subgroup, comprising seven MS and six control myelin samples, after 6 hours (*P* = 0.0023 for percentage and *P* = 0.0082 for total uptake) and 24 hours (*P* = 0.0082 for percentage and *P* = 0.0023 for total uptake) incubation (Table 3 and Additional file 3D). Analysis of the same age-matched myelin samples revealed a significantly enhanced total uptake of MS myelin by primary microglia (*P* = 0.0496). However, significance was lost within the PMD-matched myelin samples (*P* = 0.0734), probably because of a decrease in power, although a trend still remained. We concluded that MS myelin is taken up more efficiently than control myelin, irrespective of donor age and PMD.

**Table 3:** Comparison of phagocytosis of MS versus control myelin in the different matched subgroups

	**Myelin uptake**	**All donors**	**Matched for age**	**Matched for PMD**
**THP-1 (6 hours)**	**Percentage**	0.0010^b^	0.012^a^	0.0023^b^
	**GeoMFI**	0.0005^c^	0.0016^b^	0.0082^a^
**THP-1 (24 hours)**	**Percentage**	0.0006^b^	0.0004^c^	0.0082^a^
	**GeoMFI**	0.0001^c^	0.0008^b^	0.0023^b^
**Microglia (24 hours)**	**Percentage**	0.4791	0.3884	0.9452
	**GeoMFI**	0.0106^a^	0.0496^a^	0.0734

GeoMFI, geomean fluorescence intensity; PMD, postmortem delay. ^a^*P* < 0.05; ^b^*P* < 0.005; ^c^*P* < 0.0005.

Comparison in uptake of MS versus control myelin by THP-1 macrophages after 6 and 24 hours, and by primary human microglia after 24 hours, expressed as the percentage of cells that had phagocytosed myelin and as the amount of myelin taken up by the cells (geoMFI). Data were analyzed with the Mann–Whitney *U* test. *P* values are provided for of all myelin samples, as well as for age- and PMD-matched subgroups.

## Discussion

We are the first to show that myelin from NAWM of MS brain donors is taken up more efficiently than myelin from control donors by the human macrophage cell line THP-1 and by primary human microglia. Both the percentage of cells that had phagocytosed myelin and the total amount of myelin phagocytosed by the cells were significantly higher in THP-1 macrophages incubated with MS myelin compared with control myelin after 6 and 24 hours. After 6 hours, the percentage of phagocytosing cells was still increasing, indicating that factors in MS myelin influence early-stage phagocytosis. Enhanced uptake was also observed after 24 hours, at which time a plateau was reached in the percentage of cells that phagocytosed myelin. We also quantified myelin phagocytosis by primary human microglia isolated from fresh postmortem human brain tissue of 10 donors, which showed a more-linear increase in myelin phagocytosis over 72-hours. Albeit the percentage of microglia that had phagocytosed MS versus control myelin was the same, the total uptake of MS myelin was higher compared with control myelin.

The age and PMD of MS and control donors differed significantly, because myelin was isolated as donors came to autopsy. Because age and PMD correlated with myelin phagocytosis (negatively and positively, respectively), we corrected for these parameters. Comparison of myelin uptake within subgroups of retrospectively age- or PMD-matched myelin samples still revealed a significantly enhanced uptake of MS myelin in both cell types. Significance was lost only in the PMD-matched myelin samples taken up by primary microglia, but this was likely because of loss of power caused by the smaller sample size, and a trend remained detectable. We therefore conclude that phagocytosis of MS myelin is more efficient than that of control myelin, irrespective of donor age and PMD, possibly due to the changes in MS myelin already described in literature [3-5,10-12,15,16,19-21,23-26] or to other yet-unknown changes that may facilitate phagocytosis. Lipid peroxidation did not influence phagocytosis in our experiments and could thus not underlie the observed differences in uptake. It would be very interesting to identify the targets in MS myelin that cause its increased uptake. However, although the differences in uptake are significant, they are small, and the changes found previously in MS myelin are numerous. We therefore considered identifying these targets beyond the scope of this article.

The role of microglia and infiltrating blood-borne macrophages in myelin phagocytosis in MS is still debated [32]. Depletion of infiltrating macrophages abolishes neurologic symptoms in animal models of MS [33,34]. Conversely, rat and human microglia are better capable of myelin phagocytosis than macrophages *in vitro*[10,35]. This study shows that isolated primary human microglia are capable of myelin phagocytosis, but that they are slower to start phagocytosing than THP-1 macrophages. Unlike THP-1 macrophages, uptake by microglia was more linear and did not reach a plateau within the time frame studied. THP-1 macrophages phagocytosed either negligible or large amounts of myelin, as can be concluded from the two distinct populations detected after myelin incubation with flow cytometry. In contrast, microglia showed a more-gradual shift in pHrodo signal, indicating that most cells had taken up myelin. Of note, primary microglia took up a large amount of myelin despite being much smaller than THP-1 macrophages.

These results obtained with freshly isolated human microglia indicate that microglia are indeed very capable of contributing to myelin phagocytosis in MS, likely together with infiltrating macrophages. Previous research has shown that microglia isolated from NAWM of MS patients show an increased CD45 surface expression immediately *ex vivo* compared with microglia from control donors, indicating that microglia from MS patients are alerted [30]. It would be interesting to investigate in a future study whether MS microglia also differ in their phagocytic capacity for myelin. Of note, an increase in surface CD45 expression *per se* does not indicate a higher phagocytic ability. In our populations, the initial activation state did not correlate with myelin phagocytosis, which also makes it unlikely that the neurologic disease of the microglia donors that we investigated here has influenced the results.

Interestingly, we found a dual age effect. Myelin isolated from younger donors was phagocytosed more efficiently than myelin isolated from older donors. Conversely, older microglia increased their myelin phagocytic ability as myelin phagocytosis showed a positive trend with the age of microglia. Previous research showed that myelin degradation increased during aging in monkeys [36]. Furthermore, the incidence of dystrophic microglia, showing abnormal morphology, increases during aging [37]. Functionally, microglia from older mice phagocytosed less amyloid β than did microglia from younger mice in an Alzheimer disease model, which could explain why amyloid β accumulates in the brain with age [38]. It is generally accepted that microglia become slightly more proinflammatory during aging, which is usually associated with diminished phagocytic capacity [39,40]. However, a very recent study suggests that microglia in mice show a shift toward an alternative neuroprotective priming state to compensate for the increased neuronal cell injury and death and subsequent neurotoxicity during aging [41]. We found a trend toward a positive correlation between the age of microglia donors and myelin phagocytosis, indicating that the change in phagocytic capacity during aging depends on the nature of the antigen. Collectively, these data show that aging enhances the myelin phagocytic capacity of microglia but reduces the myelin susceptibility for phagocytosis. It remains to be shown whether the latter, in conjunction with multiple sclerosis-specific changes, contributes to the fact that MS usually establishes in young adults [42] or even children [43].

## Conclusions

We show that MS-derived myelin is taken up more efficiently than control myelin, indicating that changes in the myelin trigger phagocytosis. Although the difference is relatively small, it may be highly relevant, because myelin was isolated from the NAWM of MS donors where microglia are not actively demyelinating, and myelin still visually appears unaffected. Importantly, this indicates that the phagocytosis-inducing changes might already occur early in disease pathology. Lesion onset is thought to start with the activation and accumulation of microglia [44,45], possibly reacting to changes in myelin.

The important next step will be to identify the changes in the myelin from MS patients that promote phagocytosis and investigate myelin-induced changes in myeloid cells that facilitate myelin uptake in MS.

## Consent

Permission was obtained from donors for brain autopsy and the use of tissue and clinical information for research purposes.

## Abbreviations

7AAD: 7-amino-actinomycin D; BCA: bicinchoninic acid; CSF: cerebrospinal fluid; DMSO: dimethylsulfoxide; EAE: experimental autoimmune encephalomyelitis; geoMFI: geomean fluorescence intensity; HSP: heat-shock protein; MBP: myelin basic protein; MDA: malondialdehyde; MOG: myelin oligodendrocyte glycoprotein; MS: multiple sclerosis; NAWM: normal-appearing white matter; oxLDL: oxidized low-density lipoprotein; PAD: peptidylarginine deiminase; PLP: proteolipid protein; PMA: phorbol myristate acetate; PMD: postmortem delay; SR: scavenger receptor; TBA: 2-thiobarbituric acid; TBARS: thiobarbituric acid-reactive substance; TCA: trichloroacetic acid.

## Competing interests

The authors declare that they have no competing interests.

## Authors’ contributions

DAEH carried out the myelin and microglia isolations, performed the phagocytosis experiments, analyzed and interpreted all data, and drafted the manuscript. KGS carried out myelin and microglia isolations and phagocytosis experiments. MvD carried out phagocytosis experiments. JH participated in the study design and coordination, interpreted data, and helped to draft the manuscript. IH participated in the study design and coordination, interpreted data, helped to draft the manuscript, and is the group leader of the neuroimmunology research group. All authors read and approved the final manuscript.

## Supplementary Material

Additional file 1 **Microscopy of pHrodo-labeled myelin phagocytosis by primary human microglia.** Primary human microglia incubated with pHrodo-labeled myelin for 72 hours. With phase-contrast microscopy, large clumps of myelin are visible, but only phagocytosed myelin emits fluorescent signal (63× magnification). Click here for file

Additional file 2 **Trend between myelin uptake and donor age of 10 primary human microglia isolates.** Provided is the percentage of cells that had phagocytosed myelin (upper panel) and the amount of myelin taken up by the cells (geoMFI; lower panel) as the mean of all myelin samples (blue dots), the MS myelin samples (red dots), and the control myelin samples (green dots). Data were analyzed with the Spearman rank correlation test. MS, multiple sclerosis; HC, healthy control. Click here for file

Additional file 3 **Analysis of myelin uptake in age- and PMD-matched myelin samples.** (A) Age and PMD of myelin donors differed significantly between MS and control donors. (B) Both age and PMD correlated with myelin phagocytosis, provided here as the percentage of cells that had phagocytosed myelin and as the amount of myelin taken up by the cells (geoMFI). (C) Correlation between myelin uptake (geoMFI) and age (left panel) or PMD (right panel) was used to define subgroups of myelin samples that matched for age and PMD (between the lines); shown here for THP-1 macrophages after 24 hours of incubation with myelin. Open black circles are control myelin samples. Closed red circles are MS myelin samples. (D) Enhanced uptake (geoMFI) of MS myelin compared with HC myelin in age- and PMD-matched subgroups by THP-1 macrophages after 24 hours of incubation with myelin. A similar strategy was applied for analyzing myelin uptake (percentage and total amount) by THP-1 macrophages after 6 hours of incubation and by primary human microglia after 24 hours of incubation (not shown). A summary of all comparisons is provided in Table 3. Data were analyzed with the Mann-Whitney *U* test. MS, multiple sclerosis; HC, healthy control. * < 0.05; ** < 0.005; *** < 0.0005. Click here for file
