# Supplementary figures and images for: Enhanced uptake of multiple sclerosis-derived myelin by THP-1 macrophages and primary human microglia
Source: J Neuroinflammation. 2014 Mar 31;11:64. doi: 10.1186/1742-2094-11-64 (PMC4108133; doi:10.1186/1742-2094-11-64)

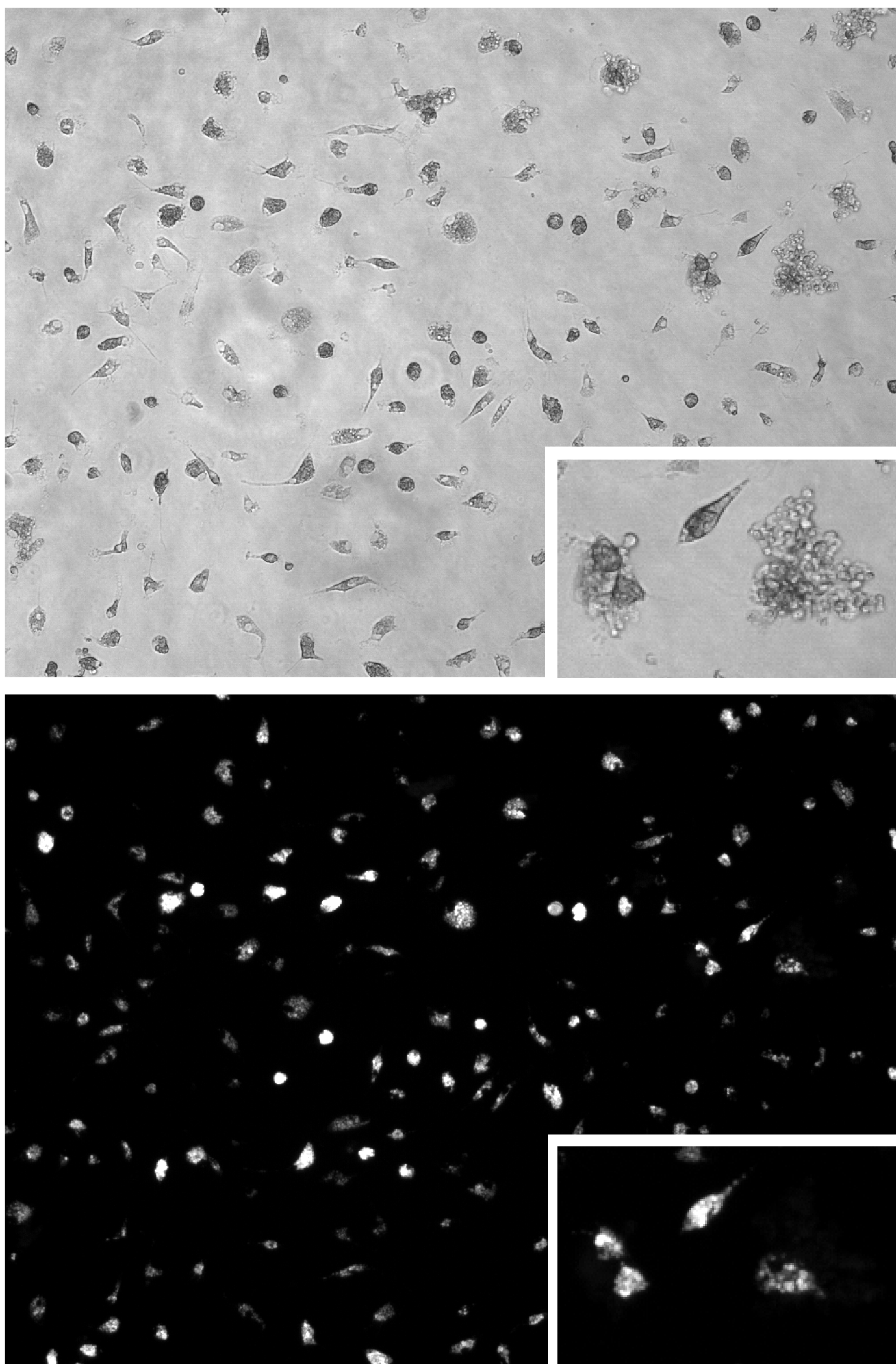

Supplement: Additional file 1 — Microscopy of pHrodo-labeled myelin phagocytosis by primary human microglia. Primary human microglia incubated with pHrodo-labeled myelin for 72 hours. With phase-contrast microscopy, large clumps of myelin are visible, but only phagocytosed myelin emits fluorescent signal (63× magnification). [file 1742-2094-11-64-S1.tiff]

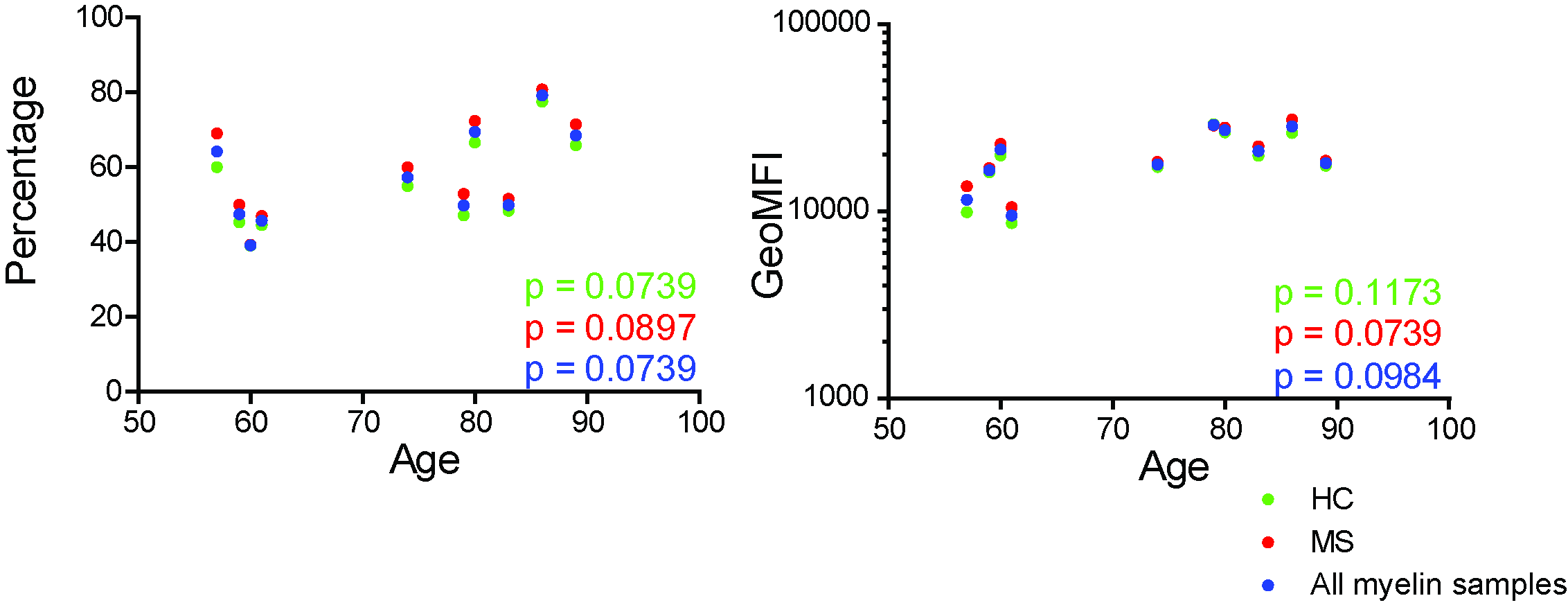

Supplement: Additional file 2 — Trend between myelin uptake and donor age of 10 primary human microglia isolates. Provided is the percentage of cells that had phagocytosed myelin (upper panel) and the amount of myelin taken up by the cells (geoMFI; lower panel) as the mean of all myelin samples (blue dots), the MS myelin samples (red dots), and the control myelin samples (green dots). Data were analyzed with the Spearman rank correlation test. MS, multiple sclerosis; HC, healthy control. [file 1742-2094-11-64-S2.tiff]

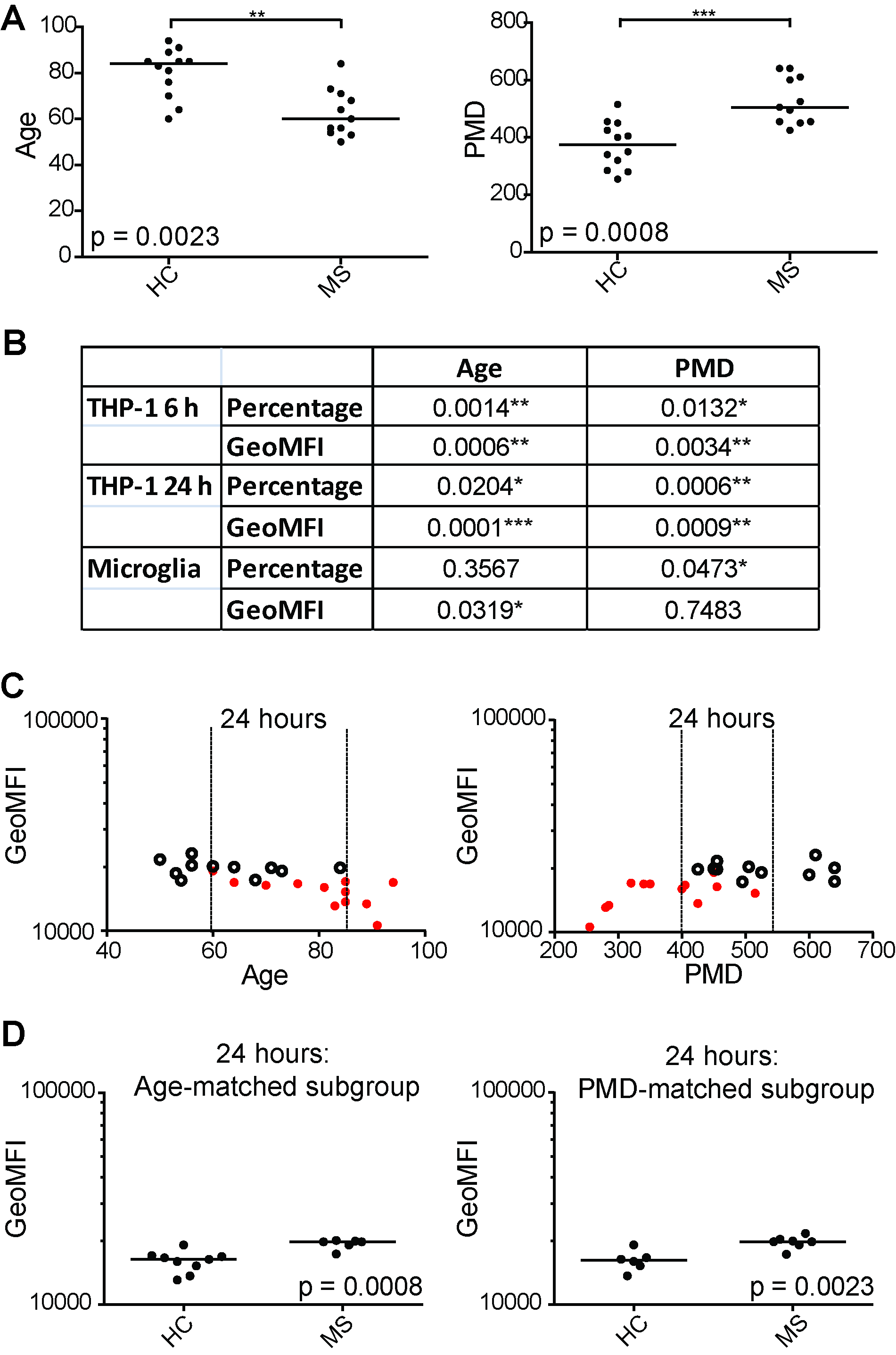

Supplement: Additional file 3 — Analysis of myelin uptake in age- and PMD-matched myelin samples. (A) Age and PMD of myelin donors differed significantly between MS and control donors. (B) Both age and PMD correlated with myelin phagocytosis, provided here as the percentage of cells that had phagocytosed myelin and as the amount of myelin taken up by the cells (geoMFI). (C) Correlation between myelin uptake (geoMFI) and age (left panel) or PMD (right panel) was used to define subgroups of myelin samples that matched for age and PMD (between the lines); shown here for THP-1 macrophages after 24 hours of incubation with myelin. Open black circles are control myelin samples. Closed red circles are MS myelin samples. (D) Enhanced uptake (geoMFI) of MS myelin compared with HC myelin in age- and PMD-matched subgroups by THP-1 macrophages after 24 hours of incubation with myelin. A similar strategy was applied for analyzing myelin uptake (percentage and total amount) by THP-1 macrophages after 6 hours of incubation and by primary human microglia after 24 hours of incubation (not shown). A summary of all comparisons is provided in Table 3. Data were analyzed with the Mann-Whitney U test. MS, multiple sclerosis; HC, healthy control. * < 0.05; ** < 0.005; *** < 0.0005. [file 1742-2094-11-64-S3.tiff]
